# A combined nucleocapsid vaccine induces vigorous SARS-CD8+ T-cell immune responses

**DOI:** 10.1186/1479-0556-3-7

**Published:** 2005-08-22

**Authors:** Ali Azizi, Susan Aucoin, Helina Tadesse, Rita Frost, Masoud Ghorbani, Catalina Soare, Turaya Naas, Francisco Diaz-Mitoma

**Affiliations:** 1 Infectious Disease and Vaccine Research Centre, Children's Hospital of Eastern Ontario Research Institute, 401 Smyth Road, Ottawa, ON, K1H 8L1, Canada; 2 Department of Microbiology and Immunology, Faculty of Medicine, University of Ottawa, Ottawa, ON, K1H 8M2, Canada

**Keywords:** Vaccine, SARS, Nucleocapsid, XIAP

## Abstract

Several studies have shown that cell-mediated immune responses play a crucial role in controlling viral replication. As such, a candidate SARS vaccine should elicit broad CD8+ T-cell immune responses. Several groups of mice were immunized alone or in combination with SARS-nucleocapsid immunogen. A high level of specific SARS-CD8+ T-cell response was demonstrated in mice that received DNA encoding the SARS-nucleocapsid, protein and XIAP as an adjuvant. We also observed that co-administration of a plasmid expressing nucleocapsid, recombinant protein and montanide/CpG induces high antibody titers in immunized mice. Moreover, this vaccine approach merits further investigation as a potential candidate vaccine against SARS.

## Introduction

The SARS epidemic had a high mortality rate as well as a huge economic impact worldwide. Treatment with antiviral drugs or an effective vaccine is not available for protection against this disease [1,2]. The SARS-CoV is a single-stranded RNA virus that has been identified as a new type of coronavirus. The genome is approximately 30 kb long and contains four structural proteins: spike, envelope, matrix and nucleocapsid in the same order as other coronaviruses [3,4]. However, the sequence analysis of SARS-CoV with other members of the coronavirus family did not show more than 20% nucleotide homology [5].

The SARS-NC gene encodes a 46 kDa protein that participates in the replication and transcription of the virus and interferes with the cell cycle of host cells [6]. Previous studies in other coronavirus members suggest that this protein is highly immunogenic and could be a good target for the design of an effective vaccine [7-10]. The expression of NC in CHO cells led to the observation that this protein folds spontaneously into viral-like particles (VLPs). These particles are effectively incorporated at several stages of the virus life cycle, including assembly, budding from cells, and receptor-binding leading to membrane fusion. The viral particles also present antigens to the immune system in a structure that mimics the infectious virion [11-13].

DNA vaccines are able to induce both humoral and cellular immune responses and have demonstrated their efficacy in several experimental models [14,15]. There are several eukaryotic vectors that express recombinant proteins efficiently. However, the uptake of antigens and its presentation are critical elements in DNA vaccination strategies. One strategy to increase the potency of DNA vaccines is to prolong the survival of antigen presenting cells (APCs), especially dendritic cells. Previous studies show that survival of dendritic cells is increased in the presence of anti-apoptotic factors such as XIAP. This approach has results in increased amounts of antigen-specific CD8+ T cells [16,17].

Specific CD8+T-cells play an important role in the control of viral infection [18-20]. Activation of specific CD8+ cells results in the secretion of inflammatory cytokines (IFN-γ and TNF-α) [21] and the synthesis of effector molecules, such as perforin and granzymes which kills infected cells, decreasing virus replication and virus load [22,23].

The present study characterizes cellular and humoral immune responses to SARS-CoV in mice receiving a DNA-NC construct alone or in combination with protein and different adjuvants. The combination of DNA-NC, protein and XIAP elicited a significant anti-SARS CD8+ T-cell response independent of CD4+ T-cell immune responses.

## Materials and methods

### Cell culture

Chinese Hamster Ovary (CHO) cells were grown at 37°C, 5% CO2 in Iscove's Modified Dulbecco's Medium (IMDM: Sigma, St. Louis, MO) and supplemented with 10% Fetal Calf Serum (FCS: Life Technologies, Grand Island, NY), 100 U/ml penicillin and 100 μg/ml gentamyin.

### Construction of DNA plasmids

Total RNA was purified using an RNeasy extraction kit (Qiagen, Mississauga, Ont) from the lung tissue of an autopsied patient who died from SARS. The full-length NC (1.2 kb) gene was amplified using specific primers (forward primer: 5'-ggatccatgtctgataatggaccc-3'; reverse primer: 5'-gaattcttatgcctgagttgaatc-3'). The amplicon was purified using the QIAquick gel extraction kit (Qiagen) and cloned into the PCR 2.1 TOPO-TA vector (Invitrogen, Burlington, Ont) according to the manufacturer's instructions. After plasmid digestion, the 1.2 kb band corresponding to the NC gene was sub-cloned into BamHI-and EcoRI sites of pVAX-1 which contains a CMV promoter for high level expression in vivo. The fragment was also sub-cloned into the pEF6-Myc/His (Invitrogen) and pQE (Qiagen) vectors. The pEF6 vector was designed to over produce recombinant proteins in mammalian cell lines and was used to establish a stable cell line by using a resistant blasticidine gene. The pQE Tri system vector was used for production of proteins in bacteria. These vectors ultimately allow for the purification of the protein by immobilized metal affinity chromatography. All expression constructs were confirmed and characterized by restriction enzymes and nucleotide sequence analysis.

### Expression of recombinant nucleocapsid protein

CHO cells and JM109 bacteria were transfected with pEF6 and pQE vectors containing NC or vector alone. In order to increase and sustain expression of the NC protein, a stable NC expressing CHO cell line was established using blasticidine-supplemented medium. Cells were harvested, sonicated and lysed in lysis buffer (25 mM Tris base, 2.5 mM Mercaptoethanol, 1% Triton-X100 and a cocktail of protease inhibitors). Cell pellets were centrifuged and the supernatant was incubated with the TALON metal resin (Clontech, Palo Alto, CA) for one hour. After incubation, the mixture of protein-resin was added to the columns and washed three times with 20 bed volumes of Tris-Cl, NaCl (PH 8). The recombinant protein was eluted with 150 mM imidazole.

To confirm the proteins, samples were mixed with Laemmli loading buffer, boiled for 5 minutes and loaded on a 10% polyacrylamide gel. The proteins were then transferred to a nitrocellulose membrane by electrophoretic transfer. The membranes were blocked with 5% dried milk in PBS-Tween 20 (PBS-T) and incubated with 1/3000 dilution of a sera from a SARS patient for three hours at room temperature. After washing with PBS-Tween, the blots were incubated with anti-human IgG-HRP conjugate (BioRad, Hercules, CA) for one hour at room temperature. After incubation, the blots were washed and incubated with ECL reagent (Santa Cruz Biotechnology, Santa Cruz, CA) for one minute and exposed to X-ray film (Kodak).

### Electron Microscopy

CHO cells transfected with either the DNA vector expressing NC or DNA vector alone were harvested and washed with PBS. Pellets were then fixed with 2% glutaraldehyde. Cells were rinsed twice in 0.1 M sodium cacodylate buffer at 4°C. The cells were then fixed with 2% Osmium Tetroxide for 2 hr at 4°C. After washing with distilled water, the cells were dehydrated with increasing concentrations of ethanol and embedded in spur resin. Thin sections were stained with uranyl acetate and lead citrate. The sections were screened by using a JEOL 1010 Transmission Electron Microscope (TEM).

### Adjuvants

CpG oligodeoxynucleotide (5'-TCCATGACGTTCCTGACGTT-3') was provided by Coley (Ottawa, ON). Montanide ISA-51 mineral oil adjuvant was purchased from Seppic Inc. (Paris, France). The pcDNA3 construct expressing 1.5 kb XIAP gene encoding an anti-apoptotic gene product was a kind gift from Dr. R.G. Korneluk [24].

### Animal Immunization

Six to eight week-old female B6/C3/F1 mice (Charles River, St. Constant, PQ) were immunized subcutaneously at the base of the tail with 50 μg of DNA construct expressing the nucleocapsid gene, 5μg of nucleocapsid protein and 50 μl of montanide ISA-51 (Seppic)/30 μg CpG (Coley), or 50 μg pcDNA3-XIAP at each vaccination. Each mouse was boosted three times, at one month intervals. Fourteen days after the last boost, the mice were sacrificed and their spleens and blood was collected for further testing or for long-term storage in cryopreservation medium.

### Antibody measurement by ELISA

96 well ELISA plates were coated overnight at 4°C with NC protein, and the wells were washed with PBS containing 0.05% Tween 20 and then blocked with 1% BSA in PBS. Serially diluted sera was added and incubated for 2 h at 37°C. The plates were washed and incubated for 2 h with a 1/2000 dilution of a peroxidase-conjugated affinity-purified rabbit anti-mouse secondary antibody (Bio-Rad, Richmond, CA). The plates were washed three times and developed with O-phenylendiamine dihydrochloride (OPD) substrate (Sigma, St. Louis, MO). The color reaction was stopped with 1N HCl and absorbance was read at 490 nm with an ELISA plate reader (Bio-Rad).

### Proliferation assay

Splenocytes from immunized mice were resuspended at 2 × 10^6 ^cells/ml in RPMI 1640 containing 10% FCS, 50 μM β-mercaptoethanol and 100 U/ml penicillin/streptomycin. A 100 μl aliquot containing 2 × 10^5 ^cells was added to each well of a 96 well plate. The NC protein (100 μl at 20μg/ml) was added to each well in triplicate. As a positive control, cells were also stimulated with phorbol 12-myristate 13-acetate and ionomycin (PMA/ION). After 72 h of culture, 1 μCi [^3^H] thymidine (Amersham, Arlington Heights, IL) was added to each well. Following 16 h of incubation, cells were harvested onto glass fibre filtermats and thymidine incorporation was measured with a Microbeta beta counter (Wallac, Turku, Finland).

### Intracellular cytokine staining

Fresh blood and splenocytes from immunized mice were cultured in IMDM media in the presence of 10 μg/ml brefeldin A (Sigma) and stimulated in vitro with the NC protein (10 μg/ml) expressed in bacteria. In every experiment, a negative control (without stimulation), positive control (PMA/ION) and an irrelevant protein (HIV-1 gp120 protein) was included to control for spontaneous production of IFN-γ. Sixteen hours after incubation, the cells were washed once (1600 rpm for 5 min) with 3 ml PBS / 2% FCS / 0.01% Azide and surface-stained for 15 min with PE-labeled Ab to mouse CD3, TC-labeled Ab to mouse CD8α or CD4 (Caltag Laboratories, Hornby, ON). The cells were washed as above, fixed and permeabilized using 100 μl each of A and B fixation-permeabilization solution (Caltag Laboratories). The cells were stained intracellularly with anti-mouse IFN-γ FITC-labeled Ab and incubated for 30 min (in the dark) at 4°C. Following washing, cells were analyzed by FACScan (Becton Dickinson, Mississauga, ON). An increase of 0.1% of IFN-gamma producing cells over the unstimulated control was considered as positive response to vaccination.

### ELISPOT assay

Multiscreen-HTS plates (Millipore, Bedford, MA) were coated with 10 μg/ml of anti-mouse IFN-γ antibody (mAb AN18, Mabtech, Mariemont, OH) in PBS over night at 4°C. The plates were then washed with PBS and blocked with IMDM containing 10% FCS and 100 U/ml penicillin/streptomycin for 1 h at room temperature. The medium were removed and 4 × 10^5 ^cell suspension (100 μl/well) including NC SARS protein expressed in bacteria (10 μg/ml) or irrelevant antigens at the same concentration were added and incubated for 30 h at 37°C. After incubation, cells were removed; plates were washed with PBS+0.05% Tween 20 and incubated with 1 μg/ml of biotinylated anti-mouse IFN-γ antibody (mAb R4-6A2-Biotin, Mabtech) for 2 hr at room temperature. After further washings, 100 μl/well of 1/2000 Streptavidin-ALP-PQ (Mabtech) in PBS+ 0.5% FCS was added and incubated for 1 hr at room temperature. The plates were washed as above and developed with 100 μl per well BCIP/NBT alkaline phosphatase (Moss Inc) for 20 minutes at room temperature. The reaction was stopped with rinsing the plates with tap water. The numbers of spots were analyzed with an ELISPOT reader.

### Statistical analysis

Results were expressed as mean ± S.D. In each experiment four animals were used per group. The t-test was applied for the statistical analysis of the data. The *p *value equal to or less than 0.05 was considered significant.

## Results

### Construction of the DNA vectors and expression of SARS-nucleocapsid protein in mammalian and bacteria cells

**Figure 1:**
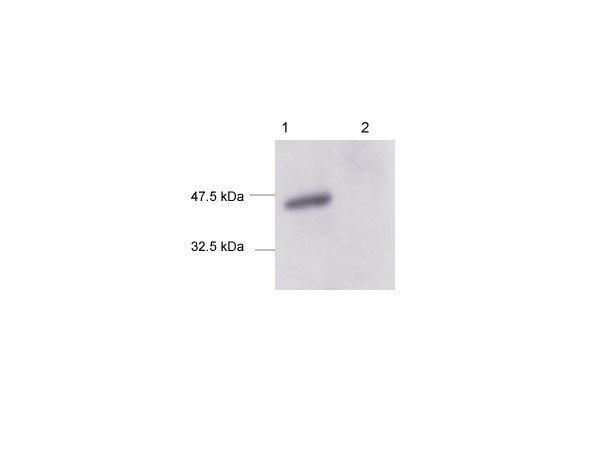
Western blot analysis of recombinant SARS-CoV-NC protein. Lane 1: purified protein from JM 109 cells transfected with pQE vector encoding nucleocapsid gene. Lane 2: represents cells transfected with the vector alone. The blot was probed with sera from a SARS patient.

To increase the potency of the specific immune response, the full-length NC was amplified by RT-PCR and ligated into plasmid pVAX-1 under the control of the human cytomegalovirus promoter. For the expression and purification of the recombinant NC protein in CHO and bacteria cells, the amplified NC gene was also sub-cloned into pEF6-Myc/His and pQE-Tri system vectors. To express NC protein, CHO cells and E.coli (JM109) were transfected with pEF6 and pQE vectors encoding the NC gene, respectively. To increase the yield of the recombinant protein, a stable CHO cell line was created using a selective resistant blasticidine gene, allowing for efficient purification of the recombinant protein. Cells were harvested, lysed and the recombinant proteins were purified according to standard methods. The expression of the NC protein in transfected cells was verified by western blotting (Fig 1) and immunofluorescence staining of CHO cells infected with the vector-NC or the vector alone. Antibody raised in rabbits to the NC protein expressed in bacteria reacted strongly in the perinuclear region of the SARS-NC-CHO cell line (data not shown).

### The assembly of NC protein into virus like particles (VLPs)

**Figure 2:**
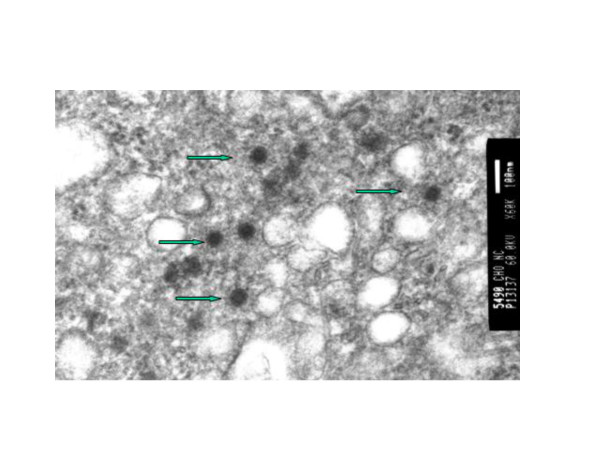
Production of viral-like particles shown by electron microscopy. The CHO cells were transfected with DNA-NC or vector alone. Arrows indicate VLPs in the transfected cell lines.

The CHO cells transfected with pEF6-NC or vector alone were examined by transmission electron microscopy. We observed bundles of VLP of the same morphology as wild type particles both inside and outside cells infected with pEF6-NC. However, neither the mock-transfected cells nor the cells transfected with the vector alone showed viral-like particles (Fig 2). These observations demonstrate that our construct expressing the NC protein synthesised sufficient protein within infected cells to facilitate the formation of VLPs.

### Detection of antibody titer in mice immunized with the candidate vaccine combinations

**Figure 3:**
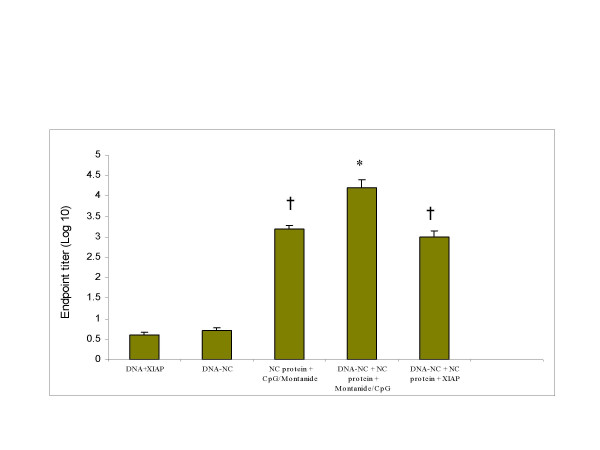
Antibody titers were determined in mice (n = 4) two weeks after the last immunization. The 96-well plates were coated with SARS-NC protein and mouse sera were serially diluted in wells for the endpoint titration of anti-NC antibody. Results are shown as mean concentration ± S.D. The symbol * indicates a significant difference (*P *= 0.01–000.1) compared with all other groups. The symbol † indicates a significant difference (*P *≤ 0.001) when compared to animals immunized with DNA+XIAP and DNA-NC alone.

In order to analyze the antibody titer against NC, five groups of mice were primed and boosted with SARS-nucleocapsid immunogen alone or in combination. Two weeks after the last boost, sera were collected and antibody titer was measured by ELISA. The group received protein and montanide/CpG showed a higher mean IgG antibody titer compared to the group receiving vector DNA+XIAP and DNA-NC alone. This group (NC protein + montanide/CpG) also showed a slightly higher antibody titer compared to the group received DNA-NC + NC protein and XIAP. However, the highest SARS-CoV-specific antibody response was detected in mice immunized with a combination of DNA-NC, protein and montanide/CpG (Fig 3).

### Combination of DNA, recombinant protein and XIAP induce higher level of CD8+T-cell immune responses

**Figure 4:**
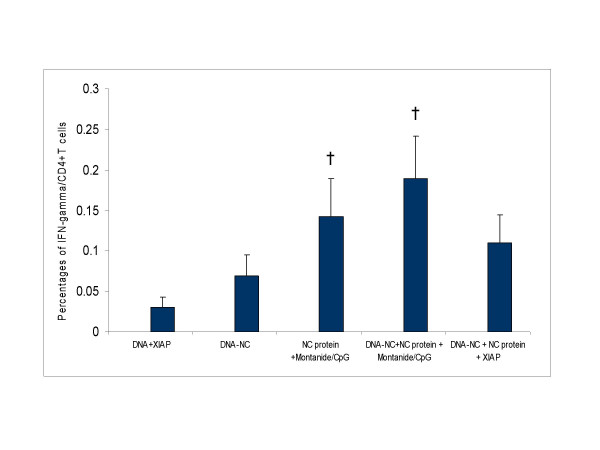
SARS-CoV-NC specific CD4+ T cell responses in mice immunized with the candidate vaccines. Fresh peripheral blood cells were cultured, stimulated with NC protein and stained for CD4, CD3 and IFN-γ. Flow cytometry was used to analyse the NC-specific CD4+T cell response. A negative control (without stimulation) and a positive control (phorbol myristate acetate + ionomycin) were included to control for the spontaneous production of IFN-γ(data not shown). Results are shown as mean ± S.D. The symbol † indicates a significant difference (*P *< 0.05) compared with the control group (DNA+XIAP).

To assess whether vaccination with nucleocapsid increases cell-mediated immune responses, splenocytes and fresh blood from immunized mice was retrieved, stimulated, and stained for surface CD4 and CD8+T cells as well as intracellular interferon gamma. The level of IFN-γ producing CD4+ T cell in fresh blood (Fig 4) and splenocytes (data not shown) from immunized mice did not demonstrate a significant CD4+T cell response against the SARS-NC protein. However, the group receiving DNA-NC, protein and montanide/CpG demonstrated higher levels of IFN-γ producing CD4+ T cells.

Splenocytes were also stimulated with NC protein, and CD4 lymphocyte proliferation was performed with tritiated thymidine. However, a high T-cell proliferation was not detected with this assay (data not shown).

**Figure 5:**
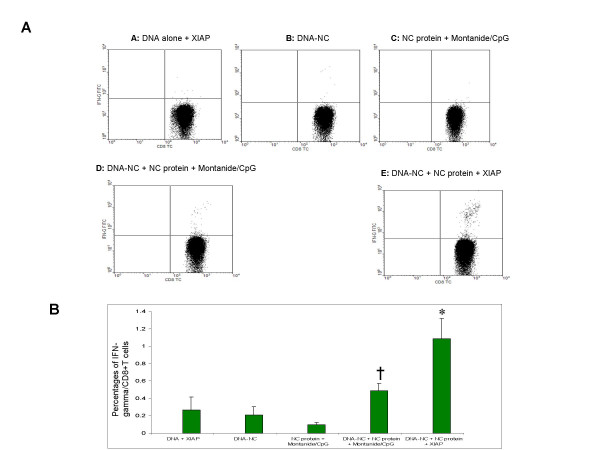
SARS-CoV-NC specific CD8+ T cell responses in mice immunized with the candidate SARS vaccines. Fresh peripheral blood cells from immunized mice were stimulated with various antigens and stained for CD8, CD3 and IFN-γ with labeled monoclonal antibodies. After staining, flow cytometry was used to analyze the NC-specific CD8+ T cells. A negative control (without stimulation) and a positive control (phorbol myristate acetate + ionomycin) were included to control for the spontaneous production of IFN-γ. Cells were also stimulated with an irrelevant protein, HIV-1 gp120 (data not shown). A: Dot plots show results from individual representative animals from each group of mice. B: Results are shown as mean ± S.D. The symbol * indicates a significant difference (*P *= 0.01–000.1) compared to all other immunized groups. The symbol † indicates a significant difference (*P *= 0.026) compared to the control group.

Cell-mediated immune responses were evaluated by intracellular cytokine staining. The group receiving DNA-NC + NC protein and Montanide/CpG elicited higher levels of CD8+T-cells to nucleocapsid in comparison to groups receiveing DNA-NC or NC protein plus adjuvant. However, the highest NC-specific CD8+T-cell response was detected in both splenocytes (data not shown) and fresh blood (Fig 5) in mice that received the DNA construct, recombinant NC protein and adjuvant XIAP.

**Figure 6:**
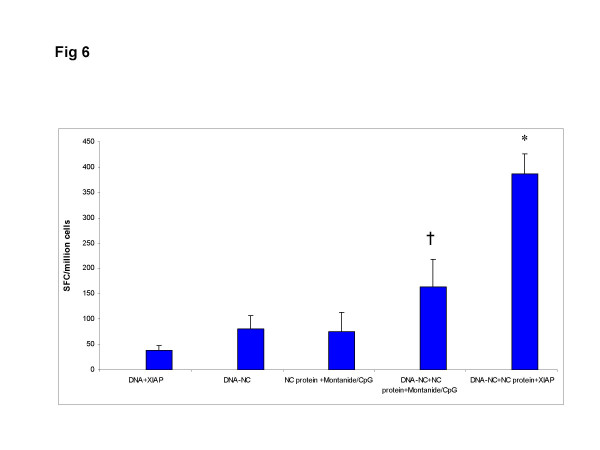
The number of IFN-γ producing cells was measured by an ELISPOT assay. The plates were coated with an anti-mouse IFN-γ antibody. The cells were cultured in the presence of recombinant NC protein or an irrelevant antigen (gp120 protein). NC-specific IFN-γ were detected as described in *Materials and Methods*. The mean ± S.D. is shown for each group. The symbol * indicates a significant difference (*P *= 0.01–0.001) between the indicated group and all other immunized groups. The symbol † indicates a significant difference (*P *< 0.05) between the indicated group and the control group (DNA+XIAP).

To confirm the results obtained by intracellular cytokine staining, we performed an IFN-γ ELISPOT assay to measure NC-specific T-cell responses of splenocytes from immunized mice. The groups DNA+XIAP, DNA-NC and NC protein + montanide/CpG did not show a high number of spot forming cells (SFC). Potent IFN-γ responses were observed in mice immunized with combination of DNA-NC+NC protein and adjuvants (Fig 6). However, following substitution of adjuvant montanide/CpG with XIAP, SFCs were more than two fold higher (*p *= 0.01). Although, IFN-γ may be produced by both antigen-stimulated CD4+ and CD8+ T cells, most likely the observed IFN-γ response was generated by effector CD8+ T-cells, since flow cytometry demonstrated CD8+ T cells as the main producers of IFN-γ in this study.

## Discussion

The SARS epidemic is currently under control. However, the absence of an effective therapeutic agent against this lethal virus, compounded by the threat of its re-emergence, has triggered research efforts to develop an effective vaccine. Previous studies indicate that the spike protein is responsible for the binding of the virus to angiotensin-converting enzyme 2 (ACE2) [25-27]. The spike protein contains epitopes that might elicit neutralizing antibodies in the host species thus making it a good target for vaccine development against SARS [28-31]. However, mutation of this protein could affect the virulence by allowing the virus to escape from specific immune response[32,33]. Other research groups have made efforts to develop vaccines based on viral nucleocapsids since these viral proteins have conserved regions. Milich and McLachlan showed that the viral nucleocapsid contains T-cell dependent and independent epitopes. Nude (athymic) mice immunized with HBV-nucleocapsid alone develop high titers of IgM, IgG2a and IgG2b antibodies which are the predominant antibodies in Th1 responses [34]. There is evidence that the specific structure folding of viral nucleocapsids is responsible for its high immunogenicity [35].

The success of immunization depends on several factors, such as type of antigen, route of administration and usage of adjuvants. Mittal et al. showed [36] that mice immunized intramuscularly, intraperitoneally or subcutaneously have higher antibody titers than mice immunized orally or intranasally. In this study, mice were immunized subcutaneously as this route of administration has been used successfully in the past [37-39].

We promoted the immune responses with adjuvants montanide ISA-51/CpG or XIAP. Montanide is a mineral oil based adjuvant that increases the immune response non-specifically[40,41]. It has been tested in clinical trials and it has a good reactogenicity profile, making it an ideal adjuvant for human use. [42-44]. In an HIV vaccine candidate study, we showed that montanide can induce strong antibody titers against HIV-1 structural genes (gp120, gag and pol). CpG is also among the most frequently used experimental adjuvants; this adjuvant stimulates dendritic cells through Toll-like receptor 9 (TLR9), inducing cell maturation and enhancing antigen presentation and Th1 responses. [45-47]. The combination of montanide and CpG was investigated in light of a recent study demonstrating that this combination is more effective than the use of any of the adjuvants alone [48]. A group of mice received XIAP as adjuvant based on the finding by Kim et al. that mice immunized with DNA encoding XIAP exhibit a strong cell mediated immune response against melanoma. Kim et al. hypothesize that this strong response may be due to increased survival of dendritic cells or T cells in vivo [16,17].

Nucleocapsid has a fundamental role in the viral life-cycle and could be a potential target for enhancing the immune responses. It is also of interest as a particulate carrier for conserved CD8+T-cell epitopes that might be suitable for the development of an effective vaccine for SARS-CoV.

In order to characterize specific immune responses in our candidate SARS vaccines, we used a recombinant protein expressed in bacteria for in vitro assays to detect CD4+ and CD8+T-cell responses, while the vaccine candidates contained a recombinant protein expressed in CHO cells. Ideally, peptides are used to stimulate CD8+effector responses, however, this is not yet feasible since NC CTL epitopes are not yet characterized in this strain of mice. It is likely that VLPs are processed by antigen presenting cells and the epitopes presented in an MHC I context, as suggested by the increased CD8+ T-cell responses observed post-vaccination.

Several studies have assessed the SARS-CoV-NC protein as a candidate vaccine. For instance, Wang et al. [49] showed a low proliferative response to NC in BALB/c mice that receive a DNA vector expressing NC protein. A weak CD4+T cell response was also observed in our study. Two more studies analyzed humoral and cell-mediated immune responses in mice immunized with DNA vaccines expressing NC [50,51]. Kim at al. showed that linkage of NC protein to calreticulin increased humoral and cellular immune responses in vaccinated mice compared to mice receiving DNA-NC alone. We did not detect a high level of CD8+ T cell immune response in mice immunized with DNA-NC or NC protein alone. However, the immunogenicity of our candidate DNA vaccine encoding NC was improved with the co-administration of the recombinant nucleocapsid protein and adjuvants.

Zhu et al. show a high level of antibody titer in mice after three injections of DNA-NC. Surprisingly, we did not detect a high level of antibody titer in mice immunized with DNA-NC alone.

In summary, our results indicate that immunization with different adjuvants could influence the type of immune response. Mice that received DNA, protein and montanide/CpG showed a high level of specific antibody titer against NC. However, vaccination with combinations of DNA-NC, recombinant NC protein and XIAP may add breadth to cell-mediated immune responses. These results suggest a novel approach to produce an effective vaccine against SARS infection.

## Abbreviations

XIAP: X-linked inhibitor of apoptosis.

NC: Nucleocapsid

## References

[B1] Buchholz UJ, Bukreyev A, Yang L (2004). Contributions of the structural proteins of severe acute respiratory syndrome coronavirus to protective immunity. Proc Natl Acad Sci USA.

[B2] Spiga O, Bernini A, Ciutti A (2003). Molecular modelling of S1 and S2 subunits of SARS coronavirus spike glycoprotein. Biochem Biophys Res Commun.

[B3] Lu H, Zhao Y, Zhang J (2004). Date of origin of the SARS coronavirus strains. BMC Infect Dis.

[B4] Egloff MP, Ferron F, Campanacci V (2004). The severe acute respiratory syndrome-coronavirus replicative protein nsp9 is a single-stranded RNA-binding subunit unique in the RNA virus world. Proc Natl Acad Sci USA.

[B5] He R, Leeson A, Ballantine M (2004). Characterization of protein-protein interactions between the nucleocapsid protein and membrane protein of the SARS coronavirus. Virus Res.

[B6] Surjit M, Liu B, Kumar P, Chow VT, Lal SK (2004). The nucleocapsid protein of the SARS coronavirus is capable of self-association through a C-terminal 209 amino acid interaction domain. Biochem Biophys Res Commun.

[B7] Wege H, Schliephake A, Korner H, Flory E, Wege H (1993). An immunodominant CD4+ T cell site on the nucleocapsid protein of murine coronavirus contributes to protection against encephalomyelitis. J Gen Virol.

[B8] Wege H, Schliephake A, Korner H, Flory E, Wege H (1993). An immunodominant CD4+ T cell site on the nucleocapsid protein of murine coronavirus contributes to protection against encephalomyelitis. J Gen Virol.

[B9] Wege H, Schliephake A, Korner H, Flory E, Wege H (1993). Coronavirus induced encephalomyelitis: an immunodominant CD4(+)-T cell site on the nucleocapsid protein contributes to protection. Adv Exp Med Biol.

[B10] Boots AM, Benaissa-Trouw BJ, Hesselink W, Rijke E, Schrier C, Hensen EJ (1992). Induction of anti-viral immune responses by immunization with recombinant-DNA encoded avian coronavirus nucleocapsid protein. Vaccine.

[B11] Young KR, Smith JM, Ross TM (2004). Characterization of a DNA vaccine expressing a human immunodeficiency virus-like particle. Virology.

[B12] Doan LX, Li M, Chen C, Yao Q (2004). Virus-like particles as HIV-1 vaccines. Rev Med Virol.

[B13] Takamura S, Niikura M, Li TC (2004). DNA vaccine-encapsulated virus-like particles derived from an orally transmissible virus stimulate mucosal and systemic immune responses by oral administration. Gene Ther.

[B14] Pachuk CJ, McCallus DE, Weiner DB, Satishchandran C (2000). DNA vaccines–challenges in delivery. Curr Opin Mol Ther.

[B15] Davis HL, McCluskie MJ (1999). DNA vaccines for viral diseases. Microbes Infect.

[B16] Kim TW, Hung CF, Ling M (2003). Enhancing DNA vaccine potency by coadministration of DNA encoding antiapoptotic proteins. J Clin Invest.

[B17] Kim TW, Hung CF, Zheng M (2004). A DNA vaccine co-expressing antigen and an anti-apoptotic molecule further enhances the antigen-specific CD8+ T-cell immune response. J Biomed Sci.

[B18] Benito JM, Lopez M, Soriano V (2004). The role of CD8+ T-cell response in HIV infection. AIDS Rev.

[B19] Gulzar N, Copeland KF (2004). CD8+ T-cells: function and response to HIV infection. Curr HIV Res.

[B20] Zhu F, Eckels DD (2002). Functionally distinct helper T-cell epitopes of HCV and their role in modulation of NS3-specific, CD8+/tetramer positive CTL. Hum Immunol.

[B21] Noble A, Leggat JA, Inderberg EM (2003). CD8+ immunoregulatory cells in the graft-versus-host reaction: CD8 T cells activate dendritic cells to secrete interleukin-12/interleukin-18 and induce T helper 1 autoantibody. Immunology.

[B22] Renner C, Held G, Ohnesorge S (1997). Role of perforin, granzymes and the proliferative state of the target cells in apoptosis and necrosis mediated by bispecific-antibody-activated cytotoxic T cells. Cancer Immunol Immunother.

[B23] Pham CT, Ley TJ (1997). The role of granzyme B cluster proteases in cell-mediated cytotoxicity. Semin Immunol.

[B24] Vitte-Mony I, Korneluk RG, Diaz-Mitoma F (1997). Role of XIAP protein, a human member of the inhibitor of apoptosis (IAP) protein family, in phytohemagglutinin-induced apoptosis of human T cell lines. Apoptosis.

[B25] Prabakaran P, Xiao X, Dimitrov DS (2004). A model of the ACE2 structure and function as a SARS-CoV receptor. Biochem Biophys Res Commun.

[B26] Xiao X, Chakraborti S, Dimitrov AS, Gramatikoff K, Dimitrov DS (2003). The SARS-CoV S glycoprotein: expression and functional characterization. Biochem Biophys Res Commun.

[B27] Li W, Moore MJ, Vasilieva N (2003). Angiotensin-converting enzyme 2 is a functional receptor for the SARS coronavirus. Nature.

[B28] He Y, Zhou Y, Liu S (2004). Receptor-binding domain of SARS-CoV spike protein induces highly potent neutralizing antibodies: implication for developing subunit vaccine. Biochem Biophys Res Commun.

[B29] Han DP, Kim HG, Kim YB, Poon LL, Cho MW (2004). Development of a safe neutralization assay for SARS-CoV and characterization of S-glycoprotein. Virology.

[B30] Zhang H, Wang G, Li J (2004). Identification of an antigenic determinant on the S2 domain of the severe acute respiratory syndrome coronavirus spike glycoprotein capable of inducing neutralizing antibodies. J Virol.

[B31] Yang ZY, Kong WP, Huang Y (2004). A DNA vaccine induces SARS coronavirus neutralization and protective immunity in mice. Nature.

[B32] Yoo D, Deregt D (2001). A single amino acid change within antigenic domain II of the spike protein of bovine coronavirus confers resistance to virus neutralization. Clin Diagn Lab Immunol.

[B33] Wang L, Xu Y, Collisson EW (1997). Experimental confirmation of recombination upstream of the S1 hypervariable region of infectious bronchitis virus. Virus Res.

[B34] Milich DR, McLachlan A, Moriarty A, Thornton GB (1987). Immune response to hepatitis B virus core antigen (HBcAg): localization of T cell recognition sites within HBcAg/HBeAg. J Immunol.

[B35] Noad R, Roy P (2003). Virus-like particles as immunogens. Trends Microbiol.

[B36] Mittal SK, Aggarwal N, Sailaja G (2000). Immunization with DNA, adenovirus or both in biodegradable alginate microspheres: effect of route of inoculation on immune response. Vaccine.

[B37] Tobiasch E, Kehm R, Bahr U (2001). Large envelope glycoprotein and nucleocapsid protein of equine arteritis virus (EAV) induce an immune response in Balb/c mice by DNA vaccination; strategy for developing a DNA-vaccine against EAV-infection. Virus Genes.

[B38] Du DW, Jia ZS, Li GY, Zhou YY (2003). HBV DNA vaccine with adjuvant cytokines induced specific immune responses against HBV infection. World J Gastroenterol.

[B39] Cui Z, Mumper RJ (2003). The effect of co-administration of adjuvants with a nanoparticle-based genetic vaccine delivery system on the resulting immune responses. Eur J Pharm Biopharm.

[B40] Aucouturier J, Dupuis L, Deville S, Ascarateil S, Ganne V (2002). Montanide ISA 720 and 51: a new generation of water in oil emulsions as adjuvants for human vaccines. Expert Rev Vaccines.

[B41] Sanderson K, Scotland R, Lee P (2005). Autoimmunity in a phase I trial of a fully human anti-cytotoxic T-lymphocyte antigen-4 monoclonal antibody with multiple melanoma peptides and Montanide ISA 51 for patients with resected stages III and IV melanoma. J Clin Oncol.

[B42] Peter K, Men Y, Pantaleo G, Gander B, Corradin G (2001). Induction of a cytotoxic T-cell response to HIV-1 proteins with short synthetic peptides and human compatible adjuvants. Vaccine.

[B43] Lawrence GW, Saul A, Giddy AJ, Kemp R, Pye D (1997). Phase I trial in humans of an oil-based adjuvant SEPPIC MONTANIDE ISA 720. Vaccine.

[B44] Barnett PV, Pullen L, Williams L, Doel TR (1996). International bank for foot-and-mouth disease vaccine: assessment of Montanide ISA 25 and ISA 206, two commercially available oil adjuvants. Vaccine.

[B45] Jiao X, Wang RY, Qiu Q, Alter HJ, Shih JW (2004). Enhanced hepatitis C virus NS3 specific Th1 immune responses induced by co-delivery of protein antigen and CpG with cationic liposomes. J Gen Virol.

[B46] Lin L, Gerth AJ, Peng SL (2004). CpG DNA redirects class-switching towards "Th1-like" Ig isotype production via TLR9 and MyD88. Eur J Immunol.

[B47] Zhang Y, Palmer GH, Abbott JR, Howard CJ, Hope JC, Brown WC (2003). CpG ODN 2006 and IL-12 are comparable for priming Th1 lymphocyte and IgG responses in cattle immunized with a rickettsial outer membrane protein in alum. Vaccine.

[B48] Kumar S, Jones TR, Oakley MS (2004). CpG oligodeoxynucleotide and Montanide ISA 51 adjuvant combination enhanced the protective efficacy of a subunit malaria vaccine. Infect Immun.

[B49] Wang Z, Yuan Z, Matsumoto M, Hengge UR, Chang YF (2005). Immune responses with DNA vaccines encoded different gene fragments of severe acute respiratory syndrome coronavirus in BALB/c mice. Biochem Biophys Res Commun.

[B50] Kim TW, Lee JH, Hung CF (2004). Generation and characterization of DNA vaccines targeting the nucleocapsid protein of severe acute respiratory syndrome coronavirus. J Virol.

[B51] Zhu MS, Pan Y, Chen HQ (2004). Induction of SARS-nucleoprotein-specific immune response by use of DNA vaccine. Immunol Lett.

